# Intraoperative Pneumatocele Formation during Liver Transplantation for Polycystic Liver Disease: Successful Non-Operative Management

**DOI:** 10.70352/scrj.cr.25-0341

**Published:** 2025-09-02

**Authors:** Satoshi Takada, Shinichi Nakanuma, Renta Kobori, Takahiro Araki, Kazuki Kato, Abdulrahman Nasr, Ryohei Takei, Daisuke Saito, Kaichiro Kato, Mitsuyoshi Okazaki, Isamu Makino, Shintaro Yagi

**Affiliations:** 1 Department of Hepato-Biliary-Pancreatic Surgery and Transplantation, Kanazawa University, Kanazawa, Ishikawa, Japan; 2 Department of Thoracic Surgery, Kanazawa University, Kanazawa, Ishikawa, Japan

**Keywords:** pneumatocele, liver transplantation, polycystic liver disease, autosomal dominant polycystic kidney disease

## Abstract

**INTRODUCTION:**

Liver transplantation for polycystic liver disease (PLD) poses significant intraoperative risks due to the presence of a massively enlarged liver. We report a rare case of intraoperative pneumothorax and pneumatocele formation during total hepatectomy, which was successfully managed with a non-operative approach.

**CASE PRESENTATION:**

A female patient in her 40s with a history of autosomal dominant polycystic kidney disease presented with progressive liver cyst enlargement (Gigot type III, Qian classification Grade 4), which led to decreased activities of daily living and intracystic hemorrhage. The patient underwent a deceased-donor liver transplantation. During mobilization of the liver from the right side of the diaphragm, the patient experienced sudden onset of pneumothorax. Incision of the diaphragm revealed a cystic structure containing a hematoma, suggesting pneumatocele formation. The pneumatocele was not resected during the ongoing operation; instead, thoracic drainage was performed as the primary intervention. Postoperatively, no air leakage was observed, and the thoracic drain was successfully removed on POD 12. The pneumatocele, which measured approximately 10 × 10 × 7 cm, showed no signs of infection, and was monitored without additional surgical intervention. On POD 19, a fever prompted further evaluation, and CT-guided cyst aspiration for culture was performed, which revealed no evidence of infection. Acute T-cell-mediated rejection was observed on POD 27, and a steroid pulse was administered, but even after that, the pneumatocele gradually decreased in size without any signs of infection.

**CONCLUSIONS:**

A pneumatocele is an uncommon but important consideration during liver transplantation for PLD, potentially resulting from barotrauma related to abrupt changes in intrathoracic pressure during hepatectomy and mechanical ventilation. Considering the risk of infection in immunosuppressed patients, close monitoring is essential. On the contrary, surgical resection also carries the risk of pulmonary or bronchial fistulae; therefore, careful consideration is required. This case demonstrates that non-operative management with careful observation can be an effective strategy in selected patients.

## Abbreviations

ADPKD
: autosomal dominant polycystic kidney disease
CVC
: central venous catheter
IVC
: inferior vena cava
MELD
: model for end-stage liver disease
MMF
: mycophenolate mofetil
PLD
: polycystic liver disease

## INTRODUCTION

Pneumatocele, an air-filled cystic lesion within the lung parenchyma, is an uncommon but well-documented complication typically observed in pulmonary infections or trauma-related settings.^1,2)^ However, its intraoperative development, particularly during abdominal procedures such as liver transplantation, is exceedingly rare and poses diagnostic and therapeutic challenges. Liver transplantation in patients with polycystic liver disease (PLD) entails unique technical difficulties due to massive liver volume and the presence of dense adhesions with the surrounding structures, including the diaphragm.^3)^ In such settings, dissection near the abdominal diaphragmatic surface may inadvertently expose or injure the adjacent thoracic components, especially in the presence of pre-existing cystic or inflammatory changes. Several reports of peumatocele after liver transplantation for PLD,^4,5)^ have been published, but none include cases in which it developed intraoperatively. We detail the intraoperative findings, management strategies, and post-operative courses and discuss the possible mechanisms underlying this unusual complication.

## CASE PRESENTATION

**Fig. 1:**
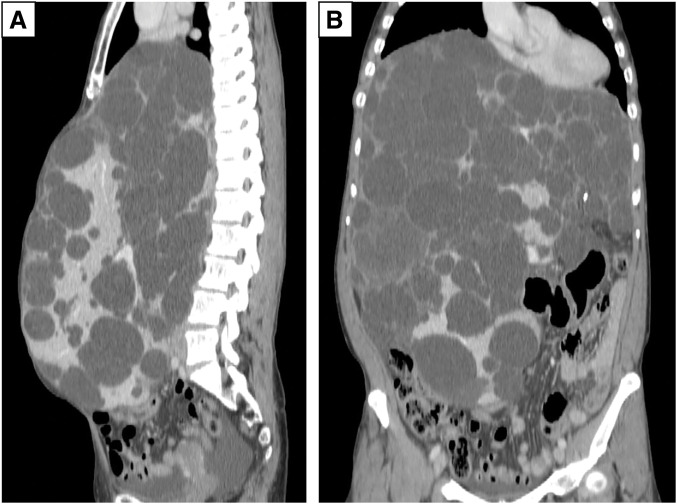
Sagittal and coronal CT images of enlarged cystic liver. (**A**) Preoperative sagittal CT image. (**B**) Coronal CT image. The abdominal cavity was filled with a cystic liver, which measured approximately 11.1 L using a 3D image analysis application.

**Fig. 2:**
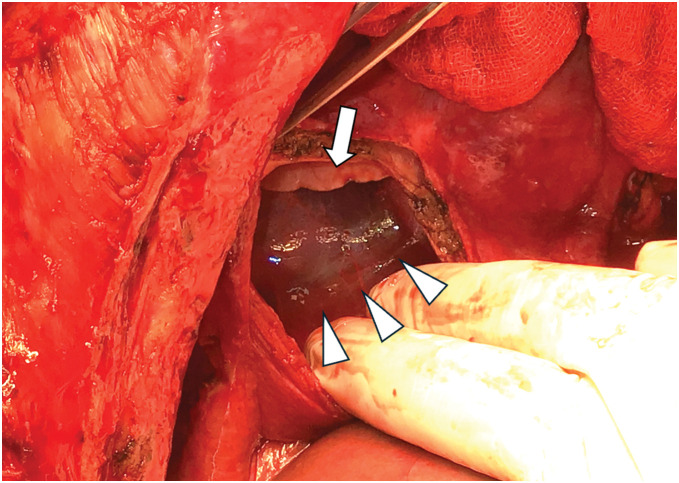
Image of a diaphragmatic incision and observation of a pneumatocele during surgery. The arrows indicate normal lungs and arrowheads indicate pneumatocele with hematoma.

**Fig. 3:**
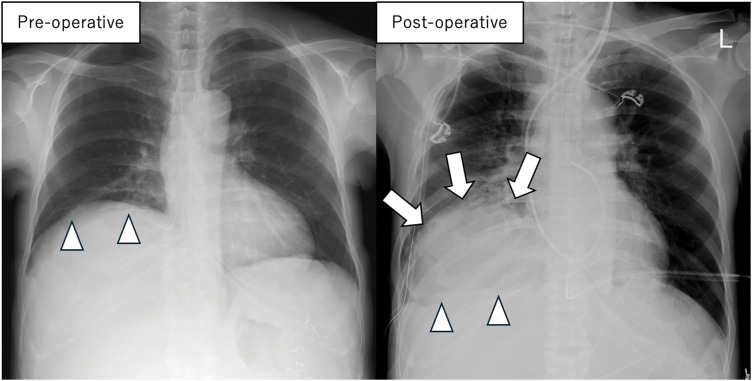
Comparison of preoperative and postoperative chest radiographs. The arrows indicate the pneumatocele and the arrowheads indicate the level of the diaphragm. Postoperatively, a large cystic structure was found to occupy the thoracic cavity.

**Fig. 4:**
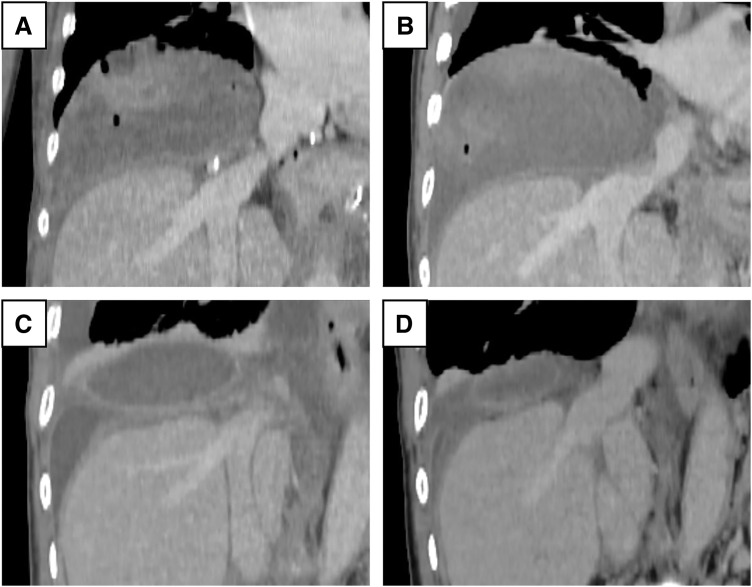
Serial coronal CT images demonstrating the natural course of the pneumatocele. (**A**) CT image obtained immediately postoperatively. (**B**) CT image at 2 weeks postoperatively. (**C**) CT image at 3 months postoperatively. (**D**) CT image at 6 months postoperatively. The pneumatocele gradually decreased in size over time, indicating a tendency toward spontaneous resolution without intervention.

A female patient in her 40s with a history of autosomal dominant polycystic kidney disease (ADPKD) presented with progressive liver cyst enlargement (Gigot type III, Qian classification grade 4) (**Fig. 1**), leading to decreased activities of daily living and intracystic hemorrhage. Preoperative estimated liver volume was 11.1 L. The MELD and MELD-Na scores were 6 and 3, respectively. The patient underwent a deceased-donor liver transplantation. The donor was a male in his 50s. After general anesthesia, a CVC and Swan-Ganz catheter were inserted and a femoro-jugular veno-venous (VV) bypass was prepared for combined excision of the giant liver along with the hepatic IVC. X-ray fluoroscopy during CVC and Swan-Ganz catheter insertion revealed no abnormal findings in the lungs. During mobilization of the liver from the right side of the diaphragm, the patient experienced a sudden onset of pneumothorax. Incision of the diaphragm revealed a cystic structure containing a hematoma, suggesting pneumatocele formation (**Fig. 2**). The pneumatocele was not resected during transplantation to avoid further bleeding. Instead, thoracic drainage was performed as the primary intervention. Methylprednisolone (500 mg) was administered at the time of graft placement and tacrolimus and mycophenolate mofetil (MMF) were initiated postoperatively. Postoperative radiographs showed that the cyst had the same height as the level of the diaphragm before surgery but had no adverse effect on the respiratory status (**Fig. 3**). The patient was extubated on POD 2. Although the air leak ceased shortly after thoracic drainage placement, the drain was maintained until POD 12 due to ongoing pleural effusion of approximately 200–300 mL per day. The pneumatocele, which measured approximately 10 × 10 × 7 cm, showed no signs of infection, and was monitored without additional surgical intervention. On POD 19, she had a fever of 39.0°C and CT-guided cyst aspiration for culture was performed revealing no evidence of infection. A pleural fluid culture was also performed, and empyema was ruled out. Acute T-cell-mediated rejection was observed on POD 27, and a steroid pulse was administered; however, the pneumatocele gradually decreased in size without any signs of infection. Steroids were only used for a short period of 6 days and then terminated. The patient was discharged on POD 61. Six months after surgery, the pneumatocele volume had shrunk to approximately 1/20th of its maximum size (**Fig. 4**).

## DISCUSSION

Here, we report a rare case of intraoperative pneumothorax and pneumatocele formation during liver transplantation for PLD. The mechanism of the pneumatocele in this case could not be determined, but it is thought to be related to surgical manipulation, intrathoracic pressure changes, and the patient’s background disease. While removing a giant liver, strong traction on the diaphragm may have damaged preformed adhesions within the thoracic cavity. In addition, a sudden drop in intrathoracic pressure may have caused barotrauma because of mechanical ventilation. Sugimura et al. reported a case of rapid development of intraoperative pneumatocele in the left lower lung lobe after left upper lobectomy in a patient with emphysema, and suggested that positive pressure ventilation may have increased the pressure on the remaining lobe.^6)^ In recent years, numerous reports of air-leak syndrome have been published associated with COVID-19 pneumonia, including pneumothorax, pneumatocele, and pneumomediastinum. Among these, reports show that invasive ventilation is an independent risk factor, along with underlying conditions such as chronic lung disease.^7)^ ADPKD itself is not associated with pulmonary fragility, although a few case reports have described its association with pulmonary cysts.^8,9)^ In our case, the pneumatocele developed rapidly during surgery, raising the possibility that a pre-existing but undetected cyst may have been present prior to transplantation. If surgical manipulation or intrathoracic pressure alone is sufficient to cause such lesions, similar events might be expected during the removal of other large hepatic tumors, such as hepatocellular carcinoma or hemangioma; however, no such cases have been reported. The waiting time for deceased donor liver transplantation in patients with ADPKD is often prolonged, during which the thoracic cavity may remain chronically compressed by the enlarged polycystic liver. Throughout this period, patients frequently experience persistent inflammatory episodes, including cyst infection and hemorrhage. These repeated insults are believed to contribute to the development of adhesions and fibrosis involving the pleura and lungs. The dynamic anatomical and physiological changes associated with liver transplantation in such patients may, therefore, predispose to rare complications such as pneumatocele formation.

Pneumatoceles are thin-walled, air-filled cystic lesions most commonly observed in pulmonary infections or trauma-related settings. Although often self-limiting, their clinical course can vary, requiring individualized management strategies based on size, location, presence of complications, and the patient’s overall clinical status. Conservative management is the 1st-line approach owing to its non-invasive nature and low risk, especially effective in stable or pediatric patients.^1)^ However, this may be insufficient in cases of lesion enlargement or other complications. Image-guided percutaneous drainage offers a minimally invasive treatment option for infected or symptomatic pneumatoceles, but carries risks such as pneumothorax, bleeding, and recurrence. In the present case, the cyst cavity was predominantly occupied by a hematoma at the time of CT-guided puncture, and only approximately 50 mL of fluid could be aspirated. We judged that drainage using a thin catheter would likely be ineffective. Based on these findings, we concluded that surgical intervention would be unavoidable if infection became evident. Therefore, we initially opted to perform aspiration alone without placing a drainage tube. Surgical intervention provides definitive management for complicated cases, but is associated with higher morbidity, particularly in critically ill or immunocompromised patients.^4)^ In this patient, a lower lobectomy would have been required in case of surgery. Given the expected need for postoperative immunosuppression and possible corticosteroid therapy, in the event of rejection, surgical intervention was deemed less favorable. As no clear evidence of infection was observed within the cystic lesion, a non-operative approach was selected. Close monitoring—including CT-guided assessment of cyst contents during febrile episodes—enabled early detection of infection and facilitated successful management without surgical intervention.

## CONCLUSIONS

Intraoperative pneumatocele formation during liver transplantation is an extremely rare event that likely results from the complex interplay among surgical manipulation, ventilation dynamics, and patient background. Our experience suggests that in the absence of a clear infection or respiratory compromise, non-operative management with close monitoring can be an effective and safe strategy. Further accumulation of similar cases is required to better understand the risk factors and optimize management strategies.

## DECLARATIONS

### Availability of data and materials

The datasets used and/or analyzed during the current study are available from the corresponding author on reasonable request.

## References

[1] Kunyoshi V, Cataneo DC, Cataneo AJM. Complicated pneumonias with empyema and/or pneumatocele in children. Pediatr Surg Int 2006; 22: 186–90. 16362309 10.1007/s00383-005-1620-5

[2] Phillips B, Shaw J, Turco L, et al. Traumatic pulmonary pseudocyst: an underreported entity. Injury 2017; 48: 214–20. 27986273 10.1016/j.injury.2016.12.006

[3] Hata K, Nishio T, Kageyama S, et al. En bloc excision of giant polycystic liver with hepatic cava and its auto-transplant caval reconstruction as a safe surgical procedure for liver transplantation. J Hepatobiliary Pancreat Sci 2022; 29: e104–7. 35305055 10.1002/jhbp.1138 PMC9790346

[4] DiBardino DJ, Espada R, Seu P, et al. Management of complicated pneumatocele. J Thorac Cardiovasc Surg 2003; 126: 859–61. 14502169 10.1016/s0022-5223(03)00367-2

[5] Yoshiyama A, Kawashima M, Nagata S, et al. Pneumatocele development after deceased-donor liver transplantation for multiple hepatic cysts due to autosomal dominant polycystic kidney disease: a case report. Surg Case Rep 2025; 11: cr.24–0005. 10.70352/scrj.cr.24-0005 PMC11937134 40144704

[6] Sugimura A, Takahashi T, Sekihara K, et al. Case of rapid formation of intraoperative pulmonary pneumatocele after lobectomy. Ann Thorac Surg 2020; 110: e331–2. 32302662 10.1016/j.athoracsur.2020.03.028

[7] Jensen AL, Litorell J, Grip J, et al. A descriptive, retrospective single-centre study of air-leak syndrome in intensive care unit patients with COVID-19. Acta Anaesthesiol Scand 2025; 69: e14582. 39936659 10.1111/aas.14582 PMC11816560

[8] Riaz H, Batool F, Hanif Mughal H, et al. Pulmonary cyst: a rare extra-renal manifestation of autosomal dominant polycystic kidney disease. J Islamabad Med Dent Col 2024; 13i(Suppl.): 574–577.

[9] Levy N, Hota P, Kumaran M. Coexisting cystic lung disease as a rare extra-renal manifestation of autosomal dominant polycystic kidney disease. Radiol Case Rep 2018; 13: 1048–52. 30228841 10.1016/j.radcr.2018.04.013 PMC6137387

